# The influence of the CRS-R score on functional outcome in patients with severe brain injury receiving early rehabilitation

**DOI:** 10.1186/s12883-021-02063-5

**Published:** 2021-01-30

**Authors:** Melanie Boltzmann, Simone B. Schmidt, Christoph Gutenbrunner, Joachim K. Krauss, Martin Stangel, Günter U. Höglinger, Claus-W. Wallesch, Jens D. Rollnik

**Affiliations:** 1 Institute for Neurorehabilitative Research, Associated Institute of the Hannover Medical School, BDH-Clinic Hessisch Oldendorf, Hessisch Oldendorf, Germany; 2 Department of Rehabilitation Medicine, Hannover Medical School, Hannover, Germany; 3 Department of Neurosurgery, Hannover Medical School, Hannover, Germany; 4 Department of Neurology, Section of Clinical Neuroimmunology and Neurochemistry, Hannover Medical School, Hannover, Germany; 5 Department of Neurology, Hannover Medical School, Hannover, Germany; 6 BDH-Clinic Elzach, Elzach, Germany

**Keywords:** Disorders of Consciousness (DoC), Coma Recovery Scale-Revised (CRS-R), Neurological Early Rehabilitation; Prognosis; Functional Outcome

## Abstract

**Background:**

The aim of the study was to determine the role of the Coma Recovery Scale-Revised (CRS-R) in the prediction of functional status at the end of neurological early rehabilitative treatment.

**Methods:**

Patients consecutively admitted to intensive or intermediate care units of a neurological rehabilitation center were enrolled in the study. Consciousness and functional status were assessed with the Coma Recovery Scale-Revised (CRS-R) and the Early Rehabilitation Barthel Index (ERBI), respectively. Both assessments were carried out weekly within the first month and at the end of early rehabilitation. Patient and clinical data were entered into a binary logistic regression model to predict functional status at discharge.

**Results:**

327 patients (112 females, 215 males) with a median age of 63 years (IQR = 53–75) and a median disease duration of 18 days (IQR = 12–28) were included. Most patients suffered from stroke (59 %), followed by traumatic brain injury (31 %), and hypoxic ischemic encephalopathy (10 %). Upon admission, 12 % were diagnosed as comatose, 31 % as unresponsive wakefulness syndrome (UWS), 35 % as minimally conscious state (MCS) and 22 % already emerged from MCS (eMCS). Of all patients undergoing complete early rehabilitative treatment (*n* = 180), 72 % showed improvements in level of consciousness (LOC). In this group, age, initial CRS-R score and gains in CRS-R score after four weeks independently predicted functional outcome at discharge.

**Conclusions:**

The study confirms the relevance of the CRS-R score for functional outcome prediction. High CRS-R scores and young age facilitate functional improvements and increase the probability to continue treatment in subsequent rehabilitation phases. Moreover, results indicate that recovery might occur over a period of time that extends beyond acute care.

## Background

Over the last 20 years, the number of survivors after severe brain damage increased due to advances in emergency medicine, intensive care and neurosurgical procedures [1]. Frequently, these patients are in an altered state of consciousness. Disorders of consciousness (DoC) include coma, the unresponsive wakefulness syndrome (UWS) and the minimally conscious state (MCS). Coma is an acute state of unarousable unresponsiveness, which usually does not last longer than a few days or weeks. Comatose patients have their eyes closed and show no behavioral signs of self-related or environmental awareness [2]. The restoration of eye-opening indicates the transition to UWS (previously known as vegetative state) [3]. UWS patients regained autonomous functions, but responses are still reflexive and not a sign of conscious processing. Some patients progress to MCS, characterized by inconsistent but reproducible signs of awareness [4]. Since MCS includes a very heterogeneous patient population, it is subdivided into MCS– and MCS+ [5]. Patients emerged from MCS (eMCS) when they regained the ability to communicate and/or use objects functionally [4].

Clinical management of patients with DoC is challenging during intensive and post-acute care and misdiagnosis is common [6, 7]. A recently published evidence-based review concluded that only the Coma Recovery Scale-Revised (CRS-R) is recommended with minor reservations to assess the level of consciousness (LOC) [8]. The accurate classification of the level of consciousness is of major importance since coma, UWS and MCS are associated with different prognoses and treatment options. For example, decisions for pharmacological treatment to improve arousal and awareness (e.g. with amantadine [9] or zolpidem [10]) or to change to palliative treatment are based upon diagnosis. In addition, pain is an important issue that has to be considered during treatment [11, 12], since MCS patients suffer from pain more frequently and may benefit from pain management [13]. Regarding prognosis, patients who are in MCS one month after the brain injury are more likely to recover within the first year than patients in UWS [14, 15]. Within both categories, patients with traumatic etiologies have a better prognosis than patients with non-traumatic etiologies [14]. In general, prognostic evaluations start as soon as patients are admitted to the intensive care unit in acute-care facilities. However, studies suggest that a considerable proportion of patients with DoC regain consciousness later, in particular during post-acute inpatient rehabilitation [16–19] or even years after the injury [20–22]. Several variables are known to be associated with prognosis (e.g., age, etiology, time since injury, functional status and level of consciousness upon admission). In post-acute settings, initial CRS-R scores [17, 19] as well as week-three [16] and week-four [18] scores are strong predictors for emergence from DoC. According to Portaccio and colleagues, patients achieved improved consciousness after a mean hospital stay of five months [17].

Most previous studies have in common that improved consciousness was used as a primary outcome measure. In the present study, however, functional status will be investigated because it is an important outcome measure in neurological early rehabilitation. The functional status at the end of early rehabilitation, measured with the Early Rehabilitation Barthel Index (ERBI), has been shown to be associated with functional status upon admission, CRS-R score upon admission, age, and time since injury [23, 24]. The objective of the present study is to identify the influence of different clinical variables collected during the first four weeks on the functional status at the end of rehabilitative treatment. In light of previous studies, we assume that the CRS-R score could play a major role.

## Methods

The study was conducted at the BDH-Clinic Hessisch Oldendorf, a large neurological rehabilitation center. In Germany, neurological rehabilitation is offered in six phases [25]: acute treatment (phase A), neurological early rehabilitation (phase B), subsequent rehabilitation (phases C and D), occupational rehabilitation (phase E) and long-term care (phase F). Phase A includes the acute treatment with physical stabilization of the patient. Phase B refers to the post-acute, multimodal treatment of functionally severely impaired patients who still require intensive medical monitoring. These patients are often provided with a tracheal cannula, are mechanically ventilated, have impaired consciousness and are at a higher risk to develop complications. Phase B treatment is carried out by a multidisciplinary team of rehabilitation specialists, including neurologists, internists, nurses, physical therapists, occupational therapists, speech therapists, respiratory therapists, neuropsychologists, and social workers. Due to the large heterogeneity of phase B patients, individualized and experience-based therapy combinations tailored to the functional and medical situation of the patient are employed. Patients are transferred to subsequent rehabilitation (phase C), when they can actively participate in therapy sessions lasting 30 minutes or more twice daily.

### Inclusion Criteria

**Fig. 1:**
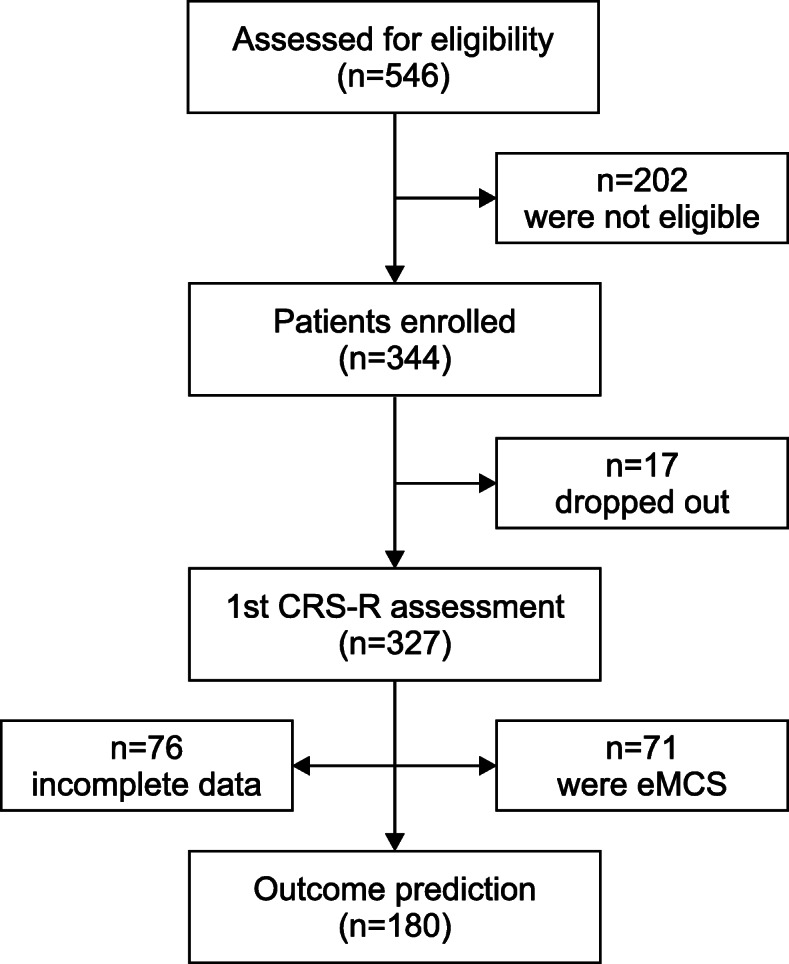
Study flow chart illustrating patient enrollment

All early rehabilitation patients (phase B) consecutively admitted to intensive care or intermediate care units between June 2018 and February 2020 were screened for eligibility (*N* = 546, see Fig. 1). Patients were included in the study, when they were at least 18 years old and suffering from stroke, traumatic brain injury or hypoxic-ischemic encephalopathy. Patients with other central (*n* = 31) or peripheral (*n* = 103) nervous system disorders, a documented history of prior brain injury (*n* = 33), a retransfer to an acute-care institution within first three days (*n* = 6), contact precautions due to colonization with multi-drug resistant bacteria (*n* = 5), a disease duration > 90 days (*n* = 11) and deficits in language comprehension due to a different first language (*n* = 13) were excluded from the study.

### Data Collection

#### Demographical and Clinical Data

Baseline data including gender, age, etiology (vascular, traumatic or anoxic), admission ward and disease duration (time between injury onset and admission to rehabilitation facility) were documented. The final medical report of each patient was retrospectively studied to obtain information about the localization of the brain injury (left, right, bilateral). In cases, when no information was available (*n* = 10), an experienced neurosurgeon was consulted. Here, the localization of the brain injury was evaluated using either a CT or a T1-weighted MRI scan, whatever was available for the respective patient. At the end of early rehabilitative treatment, length of early rehabilitative treatment and discharge disposition were determined. Moreover, patient records were screened for the occurrence of complications (i.e. hydrocephalus, pneumonia, renal insufficiency, seizure, spasticity, and urinary tract infection).

#### Functional Status

To assess the functional status, the ERBI [26] was used. The ERBI is composed of the Barthel Index (BI) [27] and the Early Rehabilitation Index (ERI) [28]. The BI, one of the most common measures in neurological rehabilitation, measures functional independence in activities of daily life through a panel of ten ordinal-scaled items. The ERI was introduced by Schönle [29] to address seven clinically important aspects among early rehabilitation patients (see Table 1 for details). The sum of the BI and the ERI results in the ERBI with a range from 325 to 100, with lower values indicating higher impairments. A prerequisite for inclusion in neurological early rehabilitation is the presence of at least one of the hard criteria for phase B (Table 1) or an total ERBI score of 30 or less. Since most early rehabilitation patients are completely dependent on nursing, and the BI does not change over a long period of time, the ERBI is considered as an useful alternative assessment instrument. In a previous study from our group, we could demonstrate that the ERBI is associated with morbidity and length of stay [26].

**Table 1:** Items of the Early Rehabilitation Barthel Index (ERBI)

Early Rehabilitation Index (ERI)	Value
Intensive care supervision	-50*
Tracheostomy tube management and supervision	-50*
Intermittent or continuous mechanical ventilation	-50*
Confusional state requiring supervision	-50*
Behavioral disturbances endangering oneself or others	-50*
Severe impairment of communication	-25
Dysphagia requiring supervision	-50*
ΣERI	-325 to 0
**Barthel Index (BI)**	
Feeding	0; 5; 10
Bathing	0; 5
Grooming	0; 5
Dressing	0; 5; 10
Bowel control	0; 5; 10
Bladder control	0; 5; 10
Toilet use	0; 5; 10
Transfers	0; 5; 10; 15
Mobility on level surfaces	0; 5; 10; 15
Stairs	0; 5; 10
ΣBI	0 to 100
**ERBI (ERI + BI)**	-325 to 100

Note. *BI* Barthel Index; *ERBI* Early Rehabilitation Barthel Index; *ERI* Early Rehabilitation Index; * If this criterion is fulfilled, the patient is assigned to phase B (early rehabilitation)

In the present study, the ERBI was rated once a week as part of regular clinical care by a team of nurses, therapists and physicians. All of them were blinded to all other study data. For the analyses, values upon admission, after four weeks of inpatient treatment and at discharge were used.

#### Consciousness

The German version of the CRS-R scale [30] was used to assess the responsiveness and to quantify the level of consciousness in each patient. The CRS-R scale consists of 23 hierarchically organized items divided into five functional subscales (auditory, visual, motor, oromotor/verbal, communication) and an arousal scale, see Table 2. The sum of subscale values forms the total CRS-R score ranging between 0 and 23, with low values reflecting reflexive behavior and higher values indicating cognitively mediated behavior. UWS is diagnosed when patients show either reflexive responses such as visual or auditory startle, localization of sounds, flexion withdrawal, abnormal posturing, oral reflexive movements or no response. In order to diagnose minimal consciousness, there must be clear evidence of at least one of the following signs indicating MCS– (visual fixation, visual pursuit, localizing noxious stimuli, automatic motor responses, object manipulation and localizing objects in space) or MCS+ (object recognition, command-following, intelligible verbalization or non-functional communication). Functional communication and/or functional object use leads to eMCS diagnosis. Since there are score ranges where patients with the same value are either diagnosed with UWS or MCS (e.g., range 7 to 9), the overall score may not be used as a diagnostic indicator.

**Table 2:** Description of subscales and items included in the CRS-R scale

Subscale		Item
Auditory function scale	4:	Consistent movement to command^a^
	3:	Reproducible movement to command^a^
	2:	Localization to sound
	1:	Auditory startle
	0:	None
Visual function scale	5	Object recognition^a^
	4	Object localization: Reaching^a^
	3	Visual pursuit^a^
	2	Fixation^a^
	1	Visual startle
	0	None
Motor function scale	6	Functional object use^b^
	5	Automatic motor response^a^
	4	Object manipulation^a^
	3	Localization of noxious stimuli^a^
	2	Flexion withdrawal
	1	Abnormal posturing
	0	None /Flaccid
Oromotor/verbal function scale	3	Intelligible verbalization^a^
	2	Vocalization/oral movement
	1	Oral reflexive movement
	0	None
Communication scale	2	Functional: accurate^b^
	1	Nonfunctional: intentional^a^
	0	None
Arousal scale	3	Attention
	2	Eye opening without stimulation
	1	Eye opening with stimulation
	0	Unarousable

Note. ^a^Item denotes MCS; ^b^Item denotes eMCS

The first CRS-R assessment was conducted three days after admission to inpatient rehabilitation. Subsequently, weekly follow-up examinations during the first month and a final examination at the end of early rehabilitative were performed. Each patient was jointly assessed by two examiners to enhance the reliability of DoC classification. The CRS-R assessments were conducted by different examiners, but one patient was always assessed by the same examiner. SBS and MB previously completed a formal training offered by the author of the German CRS-R version [30]. In addition, there was a pilot phase from January to May 2018 conducted to gain experience in the administration of the CRS-R.

### Statistical analyses

SPSS software package was used for statistical analyses. Since most of the data was not normally distributed, non-parametric statistical methods were used. Two-tailed *p* value < 0.05 was considered significant. For graphical representations, mean values and standard errors were used.

Descriptive statistics were performed using median and interquartile range ([IQR], 25th and 75th percentiles) for continuous variables and frequencies for categorical variables. Group differences were evaluated with χ^2^-tests for categorical variables and with the Mann-Whitney U test or the Kruskal-Wallis test for continuous variables. Differences between scores upon admission, after four weeks, and at discharge were tested with the Wilcoxon or Friedman test. The Spearman correlation coefficient was used to examine linear relationships. Gains in CRS-R and ERBI scores after four weeks were calculated as difference score, i.e. week four minus week one.

Functional status at the end of early rehabilitative treatment was defined as the primary outcome measure, which was dichotomized into favorable (ERBI > 30) and unfavorable outcome (ERBI ≤ 30). This cut-off was chosen because it discriminates between treatment in phase B or C. Once phase B patients reach an ERBI score of 30, they enter the subsequent rehabilitation phase C due to their persistent functional progress. With an ERBI score > 30 it is assumed, that patients can actively participate in therapy sessions lasting 30 minutes or more twice daily.

A stepwise binary logistic regression analysis was performed to determine the prognostic value of different variables for predicting functional status. Age, gender, time since injury, etiology, localization of injury, admission ward, level of consciousness (coma, UWS, MCS–, MCS+), CRS-R and ERBI scores upon admission as well as gains in CRS-R and ERBI scores after four weeks were entered as independent variables. For the model, odds ratios including confidence intervals and explained variance (Nagelkerke’s R^2^) are reported. The goodness of fit of the model was assessed with the Hosmer and Lemeshow test for logistic regression.

## Results

### Patients

Three hundred forty-four patients were enrolled in the study. Among these, 17 patients had to be excluded from further analyses due to incomplete CRS-R data (*n* = 8 patients declined to be examined in one of the follow-up assessments [these patients had a total CRS-R score ≥ 10 in the first assessment and improved in consciousness during the following days enabling them to express their unwillingness to be examined]; *n* = 6 patients were set on contact precautions due to colonization with multi-resistant bacteria, and *n* = 3 patients were discharged prematurely at own request). Thus, data of 327 patients were analyzed (112 female, 215 male). The median age of all patients was 63 years (IQR = 53–75 years). Most patients suffered from stroke (*n* = 192; 58.7 %), including ischemic insults (*n* = 92; 28.1 %) and hemorrhages (*n* = 100; 30.6 %), followed by traumatic brain injury (TBI; *n* = 101; 30.9 %), and hypoxic ischemic encephalopathy (*n* = 34; 10.4 %). The injuries were located in the left (*n* = 95; 29.1 %), the right (*n* = 116; 35.5 %) or both (*n* = 116; 35.5 %) hemispheres.

The median time between brain injury and admission to inpatient rehabilitation was 18 days (IQR = 12–28 days). Two-third were admitted to the intensive care unit (*n* = 218; 66.7 %), and one-third to an intermediate care unit (*n* = 109; 33.3 %). Upon admission, 279 patients (85.3 %) had a tracheal cannula (ERI item #2) and 167 patients (51.1 %) were on mechanical ventilation (ERI item #3). In particular, these two criteria are indicators of disease severity in neurological early rehabilitation patients. Withdrawal of the tracheal cannula and weaning from ventilation was successful in 165 patients (59.1 %) and in 147 patients (88.0 %), respectively. The median length of early rehabilitative treatment was 76 days on average (IQR = 45–108 days). The median ERBI value was − 140 (IQR=-165 to -90) upon admission and improved to -85 (IQR=-90 to -35) after four weeks (Z=-10.176; *p* < .001) and to -5 (IQR=-85 to 30) at the end of early rehabilitative treatment (Z=-14.305; *p* < .001). The higher the initial value, the higher was the ERBI after four weeks (*r* = .232, *p* < .001). The small correlation size was due to the large heterogeneity regarding the changes from week one to week four. While the ERBI score improved in many patients, the ERBI declined in 33 patients and 65 patients had no changes (± 5 points). Gains in ERBI after four weeks (Md = 50; IQR = 5-105) was higher, the lower the initial ERBI score was (*r*=-.567 *p* < .001).

At the end of early rehabilitative treatment, patients had a median number of one complication (IQR = 0–2). The most frequent complications were urinary tract infection (*n* = 90; 25.5 %), seizures (*n* = 67; 19.0 %), pneumonia (*n* = 63; 17.8 %), hydrocephalus (*n* = 51; 14.4 %), spasticity (*n* = 46; 13.0 %) and renal insufficiency (*n* = 36; 10.2 %). The majority of patients was discharged to a professional care facility (*n *= 132; 40.4 %), followed by patients (*n* = 107; 32.7 %) who continued treatment in subsequent rehabilitation phases. Other patients returned home (*n* = 28; 8.6 %) or were transferred to an acute care facility (*n* = 22; 6.7 %). Mortality was 11.6 % (*n* = 38).

### Consciousness

The first CRS-R assessment classified 141 patients as UWS (43.1 %), 115 patients as MCS (35.2 %) and 71 patients as eMCS (21.7 %). Since the CRS-R scale is not able to differentiate between coma and UWS, electronic patient records were retrospectively screened for n = 100 UWS patients without eye-opening (arousal score = 0). Among these, 40 patients were diagnosed as comatose by their attending physicians.

Of all MCS patients, 49 subjects were classified as MCS– (42.6 %) and 66 subjects as MCS+ (57.4 %). The most common items indicating MCS were “reproducible movement to command” (*n* = 62), “visual pursuit” (*n* = 53), and “object manipulation” (*n* = 48). The majority of patients classified as eMCS in the first assessment remained in this state across all assessments (*n* = 65; 91.5 %). In addition, most patients (*n* = 54; 76.1 %) showed both signs indicating eMCS (i.e. functional object use and functional communication).

The median total CRS-R score was nine in the first assessment (IQR = 4–17), and improved to 16 (IQR = 7–23) after four weeks (Z=-11.412; *p* < .001) and to 23 (IQR = 13–23) at the end of early rehabilitative treatment (Z=-11.644; *p* < .001).

**Fig. 2:**
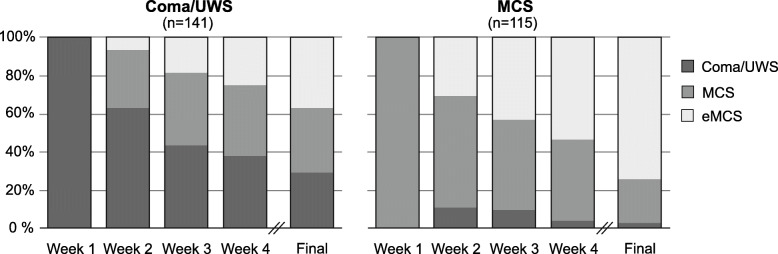
Changes of LOC categories across the first four weeks of inpatient treatment and the final assessment at the end of early rehabilitative treatment for patients identified as Coma/UWS and MCS during the first assessment

Changes of LOC categories across the first four weeks of inpatient treatment and the final assessment at the end of early rehabilitative treatment are presented in Fig. 2. 65 UWS patients (70.7 %) showed improved LOC, with 31 patients (33.7 %) transitioning to MCS and 34 patients (37.0 %) to eMCS. Among MCS patients, 65 patients (73.9 %) have emerged from MCS at the end of early rehabilitative treatment. Altogether, 130 patients (72.2 %) undergoing complete phase B treatment showed improved LOC.

### Outcome analysis

Patients diagnosed as eMCS in the first assessment (*n* = 71) were excluded from outcome analysis since this group already scores at the ceiling of the CRS-R scale, which might underestimate outcome prediction. In addition, 76 patients were excluded because they did not undergo complete early rehabilitative treatment. Reasons for missing data were transfers to acute-care institutions (*n* = 22), palliative treatment (*n* = 16), fatalities (*n* = 27) and admission to subsequent rehabilitation phases during the first four weeks (*n* = 11). Since changes in CRS-R and ERBI scores after four weeks are used as independent variables in the binary logistic regression model to predict outcome, these patients were excluded. Importantly, there were no group differences (complete vs. incomplete data) upon admission regarding age (Z=-0.67, *p* = .946), time since injury (Z=-0.142, *p* = .887), CRS-R total score (Z=-1.535, *p* = .125) and ERBI score (Z=-0.005, *p* = .996). Thus, the following analyses were performed using a sub-sample of *n* = 180 patients.

**Fig. 3:**
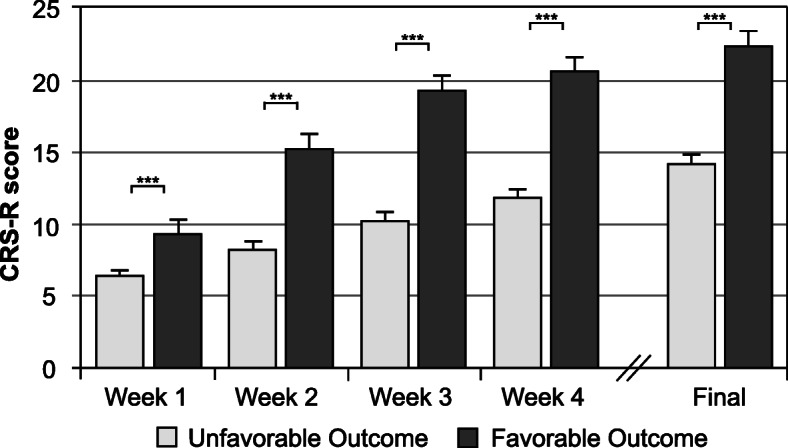
Total CRS-R scores across the first four weeks of inpatient treatment and the final assessment at the end of early rehabilitative treatment of patients with favorable and unfavorable outcome. Note. UWS = Unresponsive wakefulness syndrome; MCS = Minimally conscious state; eMCS = emerged from minimally conscious state

To predict functional outcome, patients were classified as having a favorable or unfavorable outcome at the end of early rehabilitative treatment. An ERBI > 30 (*n* = 52; 28.9 %) was considered a favorable outcome, while patients with an ERBI ≤ 30 (*n* = 128; 71.1 %) were assigned to the unfavorable outcome group. In a next step, both groups were compared (Table 3). Patients with unfavorable outcome were older (Z=-3.190, *p* < .01) and had lower CRS-R scores upon admission (Z=-3.886, *p* < .001) compared to patients with a favorable outcome. Although both groups had higher total CRS-R scores after four weeks of treatment (*p* < .001), changes were more pronounced in patients with positive outcome (Z=-2.445, *p* < .05). The comparison of individual assessments, i.e. week one to four and final assessment, revealed higher scores for the favorable outcome group across all assessments (*p* < .001), see Fig. 3. In addition, patients with an unfavorable outcome had a longer length of early rehabilitative treatment (Z=-7.709, *p* < .001). Gender, time since injury, etiology, localization of injury, admission ward, functional status upon admission, and number of complications were not related with outcome. At discharge, patients with favorable outcome showed higher gains in CRS-total score (Z=-4.922, *p* < .001) and functional status (Z=-5.886, *p* < .001).

**Table 3:** Patient characteristics presented for all patients undergoing complete early rehabilitative treatment (*n* = 180) and the two outcome groups

	Total (*n* = 180)	Favorable outcome (*n* = 52)	Unfavorable outcome (*n* = 128)
Age at event (years)	62 (51 to 75)	55 (44 to 69)	64 (53 to 78)**
Time since injury (days)	22 (12 to 28)	22 (16 to 25)	22 (11 to 29)
Male/Female (n)	119/61	35/17	84/44
Upon admission			
ERBI	-140 (-185 to -90)	-135 (-160 to -90)	-140 (-185 to -90)
CRS-R	7 (3 to 11)	9 (6 to 13)	6 (3 to 10)***
After four weeks			
ΔERBI	60 (5 to 105)	85 (30 to 160)	50 (5 to 105)*
ΔCRS-R	7 (2 to 11)	11 (7 to 16)	5 (1 to 9)***
At discharge			
Length of early rehabilitative treatment (days)	98 (67 to 115)	64 (44 to 75)	112 (87 to 132)***
Complications	1 (0 to 2)	1 (0 to 2)	1 (1 to 2)
ΔERBI	115 (55 to 160)	170 (120 to 210)	90 (35 to 135)***
ΔCRS-R	9 (4 to 14)	13 (10 to 17)	7 (2 to 12)***

Note. Values are frequencies (gender) or medians and interquartile ranges (all other variables). *ERBI* Early Rehabilitation Index; *CRS-R* Coma Recovery Scale-Revised. Mann-Whitney U test (favorable vs. unfavorable outcome): *p < .05; **p < .01; ***p < .001

**Fig. 4:**
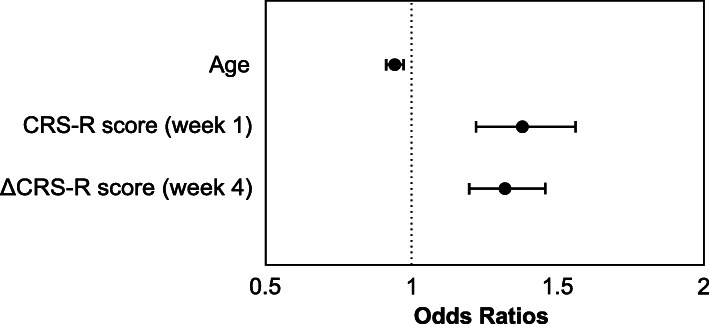
Odds ratios and confidence intervals of factors predicting a favorable outcome

All patient characteristics available upon admission and after four weeks of inpatient treatment were included in a binary logistic regression model to predict functional outcome at the end of early rehabilitative treatment. Age (OR = 0.943; CI = 0.913–0.973), total CRS-R score upon admission (OR = 1.382; CI = 1.222–1.563) and gains in CRS-R score after four weeks of treatment (OR = 1.322; CI = 1.198–1.459) proved to be independent predictors (Fig. 4). Overall, these predictors accounted for 53.5 % of the total variance of the outcome parameter (Nagelkerke’s R^2^ = 0.535). The Hosmer and Lemeshow test was not significant (Chi^2^ = 8.730, *p* = .366), confirming goodness-of-fit for the tested model.

## Discussion

The aim of the present study was to determine the influence of the CRS-R score in the prediction of functional outcome in a post-acute setting. Therefore, consciousness and functional status were monitored over a period of four weeks and at the end of neurological early rehabilitation. The majority of patients undergoing complete early rehabilitative treatment showed improved consciousness, suggesting that recovery might occur over a period of time extending beyond acute care treatment. In a binary logistic regression model with several variables collected during the first four weeks, functional outcome was independently predicted by age, initial CRS-R score and gains in CRS-R score after four weeks of inpatient rehabilitation.

In line with the results from our study, Giacino and colleagues [31] suggested that both, the initial CRS score and changes within the first month, are predictors of functional outcome. Portaccio and colleagues [18] support these findings, as they have shown that patients with improvements in CRS-R scores within the first four weeks of hospitalization have a better outcome at discharge. In another study, improved consciousness at discharge was associated with higher CRS-R scores upon admission and younger age [17]. Altogether, these studies demonstrated that a period of four weeks is a good observation period to obtain reliable prognostic information for patients with severe brain injury in different rehabilitation settings. However, it should be noted that the time between injury and admission to the rehabilitation facility differed considerably between these studies (from 18 days in our study to 1.7 months in the study of Portaccio et al. [18]). Likewise, a previous study of our group demonstrated that substantial functional improvements are to be expected within the first two months of phase B treatment [32]. These findings suggest that monitoring improvements during the first weeks of early rehabilitation might be more valid for outcome prediction than baseline assessments alone. Since different DoC are associated with different prognoses and treatment options, a reliable diagnosis is essential. For this reason, clinical scales must be able to detect subtle changes in functional abilities of the patient. Overlooking such changes might have important prognostic, therapeutic and ethical implications [8]: When behavioral signs of consciousness are not detected, albeit present, treatment interventions might not be applied or terminated too early. On the other hand, misinterpreting reflexive or other non-purposeful responses as signs of consciousness may result in overly optimistic prognoses, long or invasive treatments, and delays in adequate planning of long-term nursing care. In the worst case, a misdiagnosis may result in wrong decisions concerning life-supporting treatment. The present study confirms the role of the CRS-R for predicting favorable functional outcome at the end of neurological early rehabilitation.

Age was another independent predictor of outcome, with younger patients being more likely to have a favorable outcome. In neurological rehabilitation outcome studies, age is an important and independent prognostic factor (e.g., in stroke [33]). For phase B treatment, age has been shown to predict both functional status at discharge [23, 34] and discharge disposition [23]. In contrast to previous studies, the cause of brain damage was not related to outcome. Findings regarding etiology, however, are not consistent. Bagnato and colleagues [35] reported that patients with TBI show first signs of consciousness in more subscales and earlier than non-TBI patients. Differences might be explained by the fact that all patients were considered to be in UWS at study entry. Moreover, patients with traumatic etiologies were younger than patients with non-traumatic etiologies. Therefore, it is reasonable to assume that their better performance might be related to younger age rather than etiology [35]. Further, differences might also be attributed to different observation periods. In one study [36], etiology could predict short-term outcome six weeks after baseline, but was no longer relevant when predicting outcome at 13 weeks post-onset. Although the present study aimed to predict rather short-term outcome than long-term outcome, we also found no effect of etiology. In this context it should be noted that the German model of neurological rehabilitation is quite different from other countries, since some patients entering rehabilitation are still comatose and mechanically ventilated. Moreover, rehabilitative treatment is offered for different etiologies (vascular, traumatic, anoxic, and other injuries) under one roof instead of specialized centers for each etiology. These differences might constrain the generalizability of the results to countries with other healthcare systems. Differences between the present study and other studies might also be related to different definitions of DoC, settings (acute vs. post-acute facility), study samples (children vs. adults, traumatic vs. vascular etiology, time since injury), length of follow-up and outcome measures. Previous studies, however, confirmed results of the present studies for different settings.

While most previous studies focused on improvements of consciousness, the present study aimed to identify predictors of functional outcome. To measure functional outcome, the ERBI was used. With higher ERBI values upon admission, patients had higher ERBI values at discharge. Regarding changes of the ERBI score after four weeks of inpatient rehabilitation, a negative correlation with the initial ERBI was found. Thus, patients with low initial ERBI values made more gains in functional status than patients with initially high ratings. This relationship may be explained by the scaling of the ERI, which is part of the ERBI. In the case that a patient meets a certain criterion of the ERI, a negative value of 50 points is added to the sum score. As soon as the criterion will no longer apply, 50 points are withdrawn. This dichotomic and rough scaling of the ERI and the fact that most patients did not meet most ERI criteria at the end of early rehabilitative treatment, might explain a negative correlation.

Although age and CRS-R scores explained about 54 % of the variance of functional outcome, there is a large part of variance that cannot be explained with this model. One reason might be that some parameters known to predict outcome were not taken into account (e.g. evoked potentials, electroencephalographic patterns, and thyroid hormone levels). Moreover, it can be expected that a large part of the variance of functional outcome is explained by variables that only become available during rehabilitative treatment (e.g., presence of complications, length of rehabilitative treatment as well as type and intensity of therapeutic interventions).

Despite this, the results clearly suggest that age, the initial CRS-R score and gains in CRS-R score after four weeks independently influence functional outcome. More specifically, patients with younger age, higher CRS-R scores upon admission and major changes in CRS-R scores within the first month of inpatient treatment have a higher probability to show functional improvements. If patients improve in functional status they have greater chances to continue treatment in subsequent rehabilitation phases.

### Limitations

There are some limitations that have to be considered. First, the CRS-R scale strongly relies on language functions, although language processing is often impaired in DoC patients [37]. In addition, it is not possible to differentiate between coma and UWS when patients have their eyes closed during the assessment. Another limitation refers to the one-time assessment of the level of consciousness per day. A study by Wannez et al. (2017) shows that at least five separate CRS-R assessments over a period of two weeks are necessary to establish an accurate diagnosis [7]. In the present study, however, only one CRS-R assessment per week was conducted, since data were acquired as part of routine care. In view of the large fluctuations in patients with DoC, it is possible that some diagnoses might be incorrect. Moreover, the assessments were performed by different investigators, which could have biased the results, too. However, there are several studies proving that the CRS-R has a good inter-rater reliability (e.g., [8, 38]). The CRS-R assessment might have been influenced by medical conditions that have not been controlled. Infections, subclinical seizure activity and pain are some of the confounding factors. However, the medical staff was consulted on the patients’ condition immediately preceding the assessment. If the status was not stable enough, the assessment was postponed until the patient’s condition improved. These cases have not been documented in detail.

The ERBI is frequently used in rehabilitation settings as a measure of disability because it has a better change sensitivity than the BI in assessing severely impaired neurological patients. The main limitation, however, is the rough dichotomic scaling. Moreover, the focus is on the assessment of activities of daily life and criteria relevant for neurological early rehabilitation. In future studies, cognitive abilities should be taken into account, as is the case in the Functional Independence Measure.

## Conclusions

Overall, the present study shows that patients with severe brain injuries may regain consciousness and improve functional abilities over a period of time that extends beyond acute care. Young age, high initial CRS-R scores and major changes of CRS-R scores within the first months of inpatient treatment facilitate functional improvements, increasing the probability of continuing treatment in subsequent rehabilitation phases.

## Data Availability

The datasets supporting the conclusions of this article are available from the corresponding author on reasonable request.

## Abbreviations

BI: Barthel Index
CRS-R: Coma Recovery Scale-Revised
DoC: Disorders of Consciousness
eMCS: Emergence from MCS
ERBI: Early Rehabilitation Barthel Index
ERI: Early Rehabilitation Index
IQR: Interquartile Range
LOC: Level of Consciousness
MCS: Minimally Conscious State
TBI: Traumatic Brain Injury
UWS: Unresponsive Wakefulness Syndrome
